# Extrusion-Based Bioprinted Boron Nitride Nanotubes Reinforced Alginate Scaffolds: Mechanical, Printability and Cell Viability Evaluation

**DOI:** 10.3390/polym14030486

**Published:** 2022-01-26

**Authors:** Akesh Babu Kakarla, Ing Kong, Cin Kong, Helen Irving

**Affiliations:** 1 School of Computing, Engineering and Mathematical Sciences, La Trobe University, Bendigo, VIC 3552, Australia; A.Kakarla@latrobe.edu.au; 2 Department of Biomedical Sciences, Faculty of Science and Engineering, University of Nottingham Malaysia Campus, Semenyih 43500, Selangor, Malaysia; kong_cin@hotmail.com; 3 Department of Rural Clinical Sciences, La Trobe Institute for Molecular Sciences (LIMS), Bendigo, VIC 3552, Australia; H.Irving@latrobe.edu.au

**Keywords:** bioprinting, alginate, boron nitride nanotube, hydrogels, HEK 293T

## Abstract

Alginate (Alg) hydrogels are commonly used as bioinks in 3D bioprinting. However, one of the significant drawbacks of using Alg hydrogels is their unstable mechanical properties. In this study, a novel hydrogel-based ink composed of Alg reinforced with functionalised boron nitride nanotubes (f-BNNTs) was developed and systematic quantitative characterisation was conducted to validate its printability, physiochemical properties and biocompatibility. The printability, contact angle and mechanical test results indicated good structural stability of the scaffolds. The thermal stability of the scaffolds increased with the incorporation of f-BNNTs into Alg. Human embryonic kidney cells (HEK 293T) were seeded on the scaffolds and the cell viability was recorded for 24, 48 and 72 h. Quantitative studies showed a slight effect on toxicity with a higher concentration of BNNTs in scaffolds. The results suggest that the 3D printable f-BNNTs reinforced Alg could be used as bioink for tissue engineering applications with further studies on biocompatibility.

## 1. Introduction

Tissue engineering is an interdisciplinary field that relates to theories of both engineering and biological sciences to develop tissue implants or organs [1]. The development of tissues involves reconstructing or recovering damaged tissues [2,3]. Additionally, the tissues developed are used as replicated human tissues or organ models to investigate and test the potential effects of drugs and to better understand treatments [2,3]. Over recent decades, three-dimensional (3D) printing and bioprinting has been extensively explored for tissue engineering applications [4]. For instance, damaged tissue geometries are obtained from a patient and transferred into a machine-readable format using computer-aided design (CAD) software [4]. Later, the designed model is used to develop a native tissue model using 3D printing or bioprinting [4]. To obtain the desired structures by using a printing technique, the most crucial role is played by the materials that are used to develop the structures, support cell growth and regenerate the tissue upon printing structures.

Hydrogels made of natural or synthetic polymers have been widely employed as biomaterials, mainly in the bioprinting technique [5]. The hydrogel solution for bioprinting is often termed ink (without integration of cells) or bioink (with cells encapsulated) [1,6,7]. Most hydrogels contain a group of polymeric chains, uphold high amounts of water in their 3D network and are hydrophilic [1,8]. Due to these properties, hydrogels are compatible with cells and can produce cell-derived tissue structures through 3D bioprinting [1,8]. Recently, hydrogels such as gelatin [9], alginate [10], chitosan [11], agarose [12] and a combination of polymers [13] were studied extensively for building 3D structures using bioprinting techniques.

Alginate (Alg) is a copolymer derived naturally from brown algae [10,14,15]. It belongs to the family of polysaccharides with varying amounts of β-d-mannuronate (M) and α-l-guluronate (G) [10,14,15]. Alg hydrogels alone or in combination with other polymers have been investigated extensively as hydrogel-based inks for bioprinting due to their low toxicity [10,13,14], structural resemblance of extracellular matrices [10,13,14] and biocompatibility [10,13]. Additionally, the sol-gel transition of alginate solution induced by crosslinking with divalent ions of calcium makes them a potential candidate for bioprinting and tissue regeneration [14,16,17,18]. Studies have reported that Alg with a high M content is more immunogenic than Alg with a high G content [10,19,20]. Furthermore, researchers have found that Alg is limited in providing sufficient structural stability and mechanical properties to generate robust structures after printing [10,19,20]. To overcome these challenges, Alg composite ink solutions were developed by mixing Alg with other substances, such as gelatin [21], chitosan [22], collagen [23], polycaprolactone [24], polyvinyl alcohol [25], polylactic acid [26] and inorganic compounds such as hydroxyapatite [16] and tetraethyl orthosilicate [27]. Furthermore, the mechanical and structural properties of Alg ink can be enhanced by adding nanomaterials such as graphene [28] and carbon nanotubes [29]. There are several Alg composites that are commercially available, particularly for 3D bioprinting. These include GELXA (gelatin-alginate and gum) (Cellink, Göteborg, Sweden), alginate-cellulose nanofibrillar (RGD, Cellink, Göteborg, Sweden) and fibrin composed of nanofibrillar cellulose-sodium alginate and fibrinogen (Cellink, Göteborg, Sweden) [8,30]. However, there have been few studies on Alg reinforced with boron nitride nanotubes (BNNTs), a nanomaterial currently gaining the attention of researchers in biomedical and tissue engineering applications [31].

BNNTs are an exciting class of nanomaterials due to their unique physicochemical properties [32]. BNNTs consist of a honeycomb network with alternating boron and nitrogen atoms and are structurally similar to carbon nanotubes (CNTs) [33]. Compared to CNTs, BNNTs hold remarkably better chemical stability, electrical insulation and oxidation resistance, but display the same mechanical properties and thermal conductivity [32,34]. Similarly, BNNTs are potential materials as nanofillers for developing various polymer composites, and they have been investigated for utilisation in various fields of applications, such as electronics [32], shielding [35], sensors [36], hydrogen storage [34], drug delivery and biomedical applications [31]. However, their applications are limited due to their hydrophobic nature. To obtain a good dispersion of BNNTs in aqueous media and to increase the interfacial bonding of BNNTs with polymer matrix, BNNTs can be functionalized via several approaches [37,38,39,40]. One of them is to coat the surface of BNNTs with hydrophilic polymers [41,42]. Other approaches include introducing hydroxyl [43], amine, carboxylate [44], saline or amino groups [45] on the surface of BNNTs through oxidative conditions. Recently, BNNTs have attracted increased attention in the scientific community of tissue engineering and biomedicine. For instance, Lahiri et al. [46] demonstrated that BNNTs reinforced polylactide-polycaprolactone copolymer composite have orthopedic applications. The results indicated that incorporating BNNTs into a polymer matrix enhanced the mechanical strength and osteoblast cell viability compared to pure polymer. Another study reported that BNNTs containing gelatin-glucose scaffolds developed using electrospinning technique have tissue engineering applications [47]. Furthermore, the proliferation and attachment of human dermal fibroblast cells to scaffolds was shown to be enhanced and the degradation rate of the scaffolds was reduced by the addition of BNNTs [47]. Moreover, it is generally known that soft and hard tissue need to bear strong mechanical force under physiological conditions [29]. Hence, artificial materials that can regenerate tissues should possess good mechanical properties and be biocompatible [29]. It has been reported that the addition of nanomaterials into hydrogels to produce scaffolds with 3D bioprinting techniques has significantly improved the printability of alginate [29,48]. Considering the benefits of BNNTs, the incorporation of BNNTs into alginate represents a potentially new candidate composite material for 3D bioprinted scaffolds in tissue engineering and biomedicine.

In this study, functionalised BNNTs (f-BNNTs) reinforced Alg nanocomposites as hydrogel-based ink solutions were developed to produce a porous structure using 3D bioprinting. BNNTs were synthesised using co-precipitation and annealing processes and further purified and functionalised with hydroxyl groups. The dispersion of BNNTs in aqueous solutions was assessed before developing nanocomposite ink solution. The developed ink solution was then extruded into a grid-like porous structure to evaluate the printability. The chemical composition, mechanical and thermal properties of the produced ink with varied concentrations of f-BNNTs were evaluated. The biocompatibility of scaffolds was analysed using human embryonic kidney cells (HEK 293T). The results showed proof-of-concept for good dispersion of f-BNNTs in Alg while enhancing its physical properties. The produced hydrogel-based ink can be adapted for cell incorporation printing and tissue regeneration using 3D bioprinting.

## 2. Materials and Methods

### 2.1. Materials

Amorphous boron powder (B), iron (III) nitrate nonahydrate (Fe(NO_3_)_3_·9H_2_O), ethanol 95% grade AR and urea (CO(NH_2_)_2_) grade AR were purchased from Friendemann Schmidt Chemical (Parkwood, Australia). Ammonia gas (NH_3_, 99.5%) was purchased from BOC Gas & Gear. (Bendigo, Australia). Alginate with medium viscosity, calcium chloride (CaCl_2_), phosphate buffer solution (PBS) pH 7.4, absolute ethanol, nitric acid (HNO_3_), hydrochloric acid (HCl) and hydrogen peroxide (H_2_O_2_) were purchased from Sigma Aldrich (Melbourne, Australia). Industrial nitrogen gas (N_2_, 99%) was supplied from Coregas Pty Ltd. (Bendigo, Australia). Gibco Dulbecco’s Modified Eagle Medium (DMEM), GlutaMAX, fetal bovine serum (FBS), trypsin, Invitrogen™-ready probes for live and dead cell stains and iodonium propidium were purchased from Sigma Aldrich, Melbourne, Australia. HEK 293T cells were obtained from CellBank, Westmead, Australia.

### 2.2. Synthesis, Purification and Functionalisation of BNNTs

**Figure 1:**
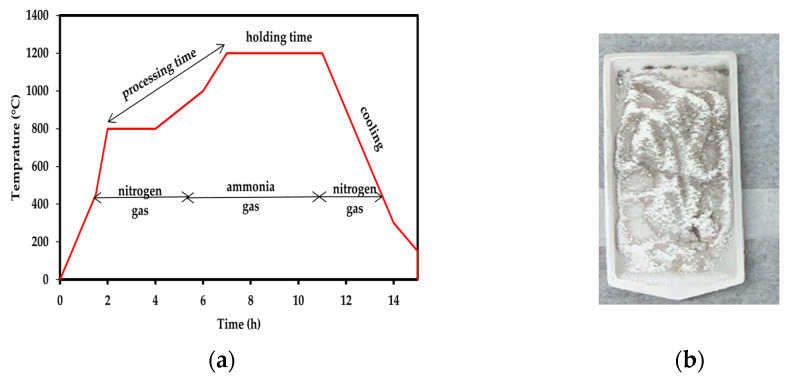
(**a**) Temperature setting for synthesis BNNTs under N_2_ and NH_3_ gas; (**b**) as-synthesised BNNTs (r-BNNTs) powder.

The BNNTs were synthesised using the method (co-precipitation and annealing process) reported by Bi et al. [49]. Briefly, a ratio of B:Fe(NO_3_)_3_·9H_2_O:CO(NH_2_)_2_ (1:0.1:2) was mixed in deionised water by stirring for 5 h at 80 °C. The mixture was kept aside for 24 h to obtain a good chemical reaction between the precursors. The precipitate that settled at the bottom of the flask after 24 h was collected. The precipitate was washed with ethanol five times and rinsed with water until the pH was 7.0. The obtained product was dried overnight in the oven at 100 °C. The dehydrated powder was later loaded into an alumina boat and placed in a tubular furnace. Prior to placing the boat here, the furnace was purged with N_2_ gas. Then, the boat was placed in the furnace and heated to 800 °C under N_2_ gas for 3 h. Afterwards, the N_2_ gas was changed to NH_3_ flow and the powder was heated at a constant temperature at 1200 °C for 5 h. Finally, the furnace was cooled to room temperature under N_2_ flow. The greyish-white powder that represents BNNTs was collected for further purification and functionalisation. The temperature setup (5 °C/min) for the synthesis of BNNTs is shown in Figure 1a, and Figure 1b shows the as-synthesised BNNTs, which are referred to as r-BNNTs.

**Figure 2:**
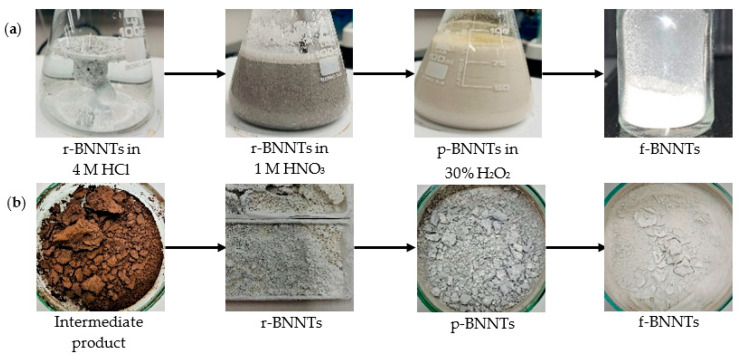
(**a**) Process of BNNTs’ purification and functionalisation; (**b**) colour change of BNNTs from intermediate to final stage.

The r-BNNTs were further purified and functionalised according to the method reported by Emanet et al. [50] (Figure 2a). Initially, the r-BNNTs were calcinated at 600 °C for 5 h. Later, the calcinated r-BNNTs were mixed with 4 M HCl solution under constant stirring at 90 °C for 4 h. The solution was centrifuged and the precipitate was mechanically stirred in 1 M HNO_3_ for 6 h. Next, the mixture was centrifuged and washed with deionised water to remove all acidic residuals until the pH was 7.0 to obtain purified BNNTs (p-BNNTs). To obtain f-BNNTs, the p-BNNTs were further refluxed in 30% H_2_O_2_ for 48 h under a constant temperature of 110 °C. Finally, the solution was centrifuged to obtain f-BNNTs with -OH groups and dried overnight at 60 °C. Figure 2b shows the colour change of the BNNTs at different stages. The f-BNNTs were further utilised for the hydrogel-based ink formulation.

### 2.3. Preparation of Alginate Reinforced with f-BNNTs Hydrogel-Based Ink

**Figure 3:**
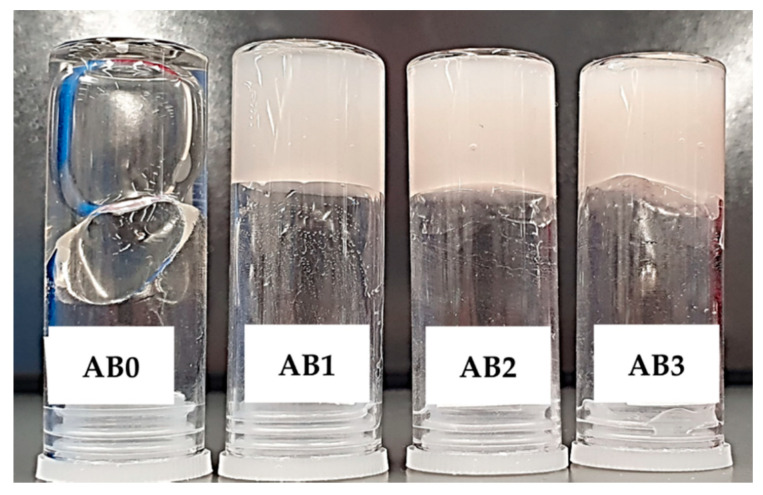
Developed hydrogel-based Alg and Alg with f-BNNTs ink for 3D bioprinting.

Hydrogel-based ink consisting of Alg and f-BNNTs reinforced Alg with varied concentrations of f-BNNTs is shown in Table 1. The Alg (5 *w*/*v*%) was dissolved in deionised water under vigorous stirring at 600 rpm. Afterwards, different concentrations of f-BNNTs were added to the Alg. The solution was sonicated using probe ultrasonication for 30 min. Finally, the solution was centrifuged at 5000 rpm for 15 min to remove bubbles in the solution, and it was stored at 4 °C. The nanocomposite hydrogel-based ink solution was prepared based on literature reports [9,28,29,51]. Hereafter, Alg and Alg reinforced with different concentrations of f-BNNTs are termed as AB0, AB1, AB2 and AB3 (Figure 3).

### 2.4. 3D Bioprinting of Alg and Alg-BNNTs Scaffolds

The scaffold models with grid-like porous structures (10 × 10 × 1 mm^3^) were printed using Cellink INKREDIBLE^+^ (Cellink, Göteborg, Sweden). The ink was loaded into a 3 mL syringe attached to a 27-gauge nozzle. Afterwards, ink was extruded into a layer-by-layer format under varied pressure (Table 1) on a petri dish at room temperature. The three-layer scaffold was printed and crosslinked immediately after printing with 100 mM CaCl_2_ solution for 15 min to obtain rigid porous scaffold structures.

### 2.5. Characterisation

#### 2.5.1. Morphology

The micrographs of r-BNNTs, f-BNNTs and alginate reinforced with f-BNNTs (AB) scaffolds were taken using a scanning electron microscope (FE-SEM, Hitachi SU7000, Tokyo, Japan). The printed scaffolds were frozen in liquid nitrogen for 30 s and images were obtained. Additionally, the morphology of BNNTs and f-BNNTs was observed under a transmission electron microscope (TEM, JEOL JEM-2100, Tokyo, Japan) to obtain high-magnification images.

#### 2.5.2. Fourier Transform Infrared Spectroscopy (FTIR)

To confirm the changes in functional groups in the synthesised BNNTs, f-BNNTs, Alg and Alg with f-BNNTs ink solutions, Fourier transform infrared spectroscopy (FTIR) spectra were recorded using Cary 630, FTIR from Agilent Technologies (Santa Clara, CA, USA), equipped with an attenuated total reflectance (ATR) accessory. Each spectrum was obtained from 32 scans at a resolution of 4 cm^−1^ from 4000 to 600 cm^−1^, using Happ-Genzel apodisation. All spectral operations were performed using MicroLab software from, V5.4, Agilent Technologies, Santa Clara, CA, USA.

#### 2.5.3. Printability

The scaffolds printed were captured using a 24-megapixel (MP) camera (Canon, EOS 200D II, Tokyo, Japan) immediately after crosslinking to obtain the printability properties. The images were processed using Fiji image processing software (ImageJ, V1.5, GNU General Public License, Bethesda, Rockville, MD, USA) to measure the strand printability, printing accuracy and printability factor (Pr).

Strand printability was measured to determine how uniform the printed strands were compared to the designed strand. Strand uniformity was measured using the following equation.

(1) Strand printablity= length of printed strand/length of designed strand

The accuracy of each composition scaffold was obtained by using Equation (2). The average of three samples (*n* = 3) was used to calculate the printing accuracy of the scaffolds.

(2) $$\mathrm{Percentage}\;\mathrm{accuracy}\;\left(\%\right)=1-\frac{\left|A_{i}-A\right|}{A}\times 100$$

*A_i_* is the initial area of the scaffold and *A* is the overall printed area of the scaffold.

Printability factor (Pr) was used to determine whether the pores matched the designed pore sizes. The printability was determined using Equation (3). Where *L* is the pore perimeter and *A* is the pore area,

(3) $$\mathrm{Pr}=L^{2}/16\times A$$

The interconnected structure would construct a perfect square shape for ideal ink printability (Pr = 1).

#### 2.5.4. Thermogravimetric Analysis (TGA)

Thermogravimetric analysis (TGA) was employed to assess the thermal stability of the f-BNNTs and AB scaffolds. The tests were performed using a TGA analyser (TGA 4000, PerkinElmer, Waltham, MA, USA). A small piece (10 to 15 mg) was cut from the printed scaffolds. The pieces were heated at 10 °C/min from 35 °C to 800 °C under a nitrogen atmosphere.

#### 2.5.5. Mechanical Properties

Compression properties were measured using a universal testing machine, Instron 5890 (Instron, Norwood, MA, USA), with a 500 N load cell. For compression, cylindrical hydrogel-based ink of 10 mm height and 10 mm diameter was prepared using a circular mould. The ink was frozen for 30 min at 4 °C. Later, the samples were taken out of the mould, crosslinked with 100 mM CaCl_2_ for 15 min and neutralised in PBS for 30 min to remove excess calcium ions. The obtained circular samples were used to analyse the mechanical properties. The stress–strain curve was plotted from compression tests of three sets of each sample, and the ultimate compressive stress was calculated from the final load applied and the cross-sectional area of the circular samples.

#### 2.5.6. Contact Angle Measurement

The AB scaffolds crosslinked with CaCl_2_ with a flat surface were prepared to measure the wettability of the hydrogel-based ink. The contact angles were calculated using Attension Theta Flex (Biolin Scientific, Västra Frölunda, Sweden) integrated with OneAttension software (V4.0, Biolin Scientific, Västra Frölunda, Sweden). The measurements were repeated three times (*n* = 3) for each ink composition.

#### 2.5.7. Cell Culture

Firstly, HEK 293T cells were cultured in DMEM media with additives (5 *v*/*v*% FBS, 2 mM/L-glutamine and 0.5 *v*/*v*% penicillin-streptomycin) in a humidified atmosphere containing 95% air and 5% CO_2_ at 37 °C. The cells were sub-cultured until a 90–95% confluence was observed. The passage levels in between 8 and 20 were used for the cell viability tests.

#### 2.5.8. Cell Viability

The viability tests of the HEK 293T cells (2.5 × 10^5^) grown after introducing AB scaffolds were evaluated with the trypan blue dye exclusion test followed by automatic measurement of live and dead cells using Countess II FL (Thermo Fisher Scientific, Melbourne, Australia) [52,53]. HEK 293T cells cultured with DMEM supplemented by additives were cultured as the control along with printed scaffolds.

The LIVE/DEAD assay (LIVE/DEAD Cell Staining Kit, Sigma Aldrich, Melbourne, Australia) was used to examine the viability of HEK 293T further after being incubated with scaffolds for 24 h. LIVE/DEAD cell staining was carried out by following the manufacturer’s instruction. Imaging was performed using a fluorescence Nikon Eclipse microscope system (cellSens V1.7, BX 71, Olympus, Tokyo, Japan). Live cells indicated by blue fluorescence and dead cells were stained by the green reactive dye.

#### 2.5.9. Statistical Analysis

Data was presented as mean ± standard deviation (SD). Statistical analysis was performed using the GraphPad Prism software (V9.0, GraphPad Software, San Diego, CA, USA). All experiments relating to the scaffold’s printability, mechanical and cell viability tests were performed using a minimum of three replicates (*n* = 3). Normality tests were performed using Shapiro–Wilk test. Statistically significant variations were defined by the Tukey pos-hoc test utilizing a one-way analysis of variances (ANOVA) for normal distribution and the Friedman analysis for non-normal distribution [54]. The *p* levels indicated with asterisks (*) set at *p* < 0.05 (*), *p* < 0.01 (**) and *p* < 0.001 (***) were considered statistical differences.

## 3. Results

### 3.1. BNNTs Morphology

**Figure 4:**
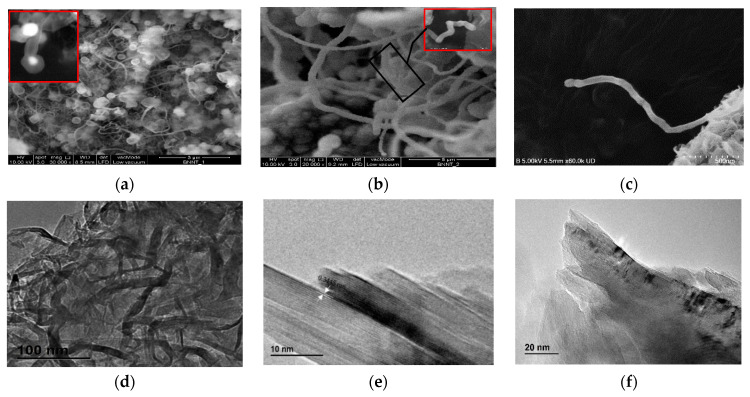
SEM images of (**a**) r-BNNTs (inset: white colour spot inside nanotubes represents the catalyst substance in nanotubes); (**b**) f-BNNTs (inset: high magnification of quasi-cylindrical nanotubes); (**c**) bamboo-like structure of f-BNNTs; TEM images of (**d**) r-BNNTs at low magnification; (**e**) r-BNNTs; (**f**) f-BNNTs.

The BNNTs were successfully synthesised using the method (co-precipitation and annealing process) reported by Bi et al. [49] at elevated temperatures (1200 °C). SEM micrographs of the as-synthesised BNNTs (r-BNNTs) with impurities are shown in Figure 4a. The inset in Figure 4a shows a cylindrical nanotube capped with catalyst substances (white colour spots) within the cavities of the nanotubes [49]. Micrographs of BNNTs after purification and functionalisation (f-BNNTs) are shown in Figure 4b. The f-BNNTs consist of both bamboo-shaped (Figure 4c) and quasi-cylindrical (Figure 4b inset)) nanotubes with white heads at the ends of the nanotubes. The particle at the end of the nanotube is a precursor that catalyses nanotube growth. The nanotubes have diameters that range from 10 to 300 nm and lengths of hundreds of microns.

The TEM image in Figure 4d shows the r-BNNTs at low magnification. The detailed structure of r-BNNTs was observed using TEM, as shown in Figure 4e. The spacing was approximately 0.34 nm of the ordered lattice fringes (0 0 2), corresponding to the plane of hexagonal boron nitride (h-BN). Thus, it was evident that it produced nanotubes composed of B and N. The TEM micrograph of f-BNNTs is shown in Figure 4f. As observed in the TEM micrograph, it was noted that some of the BNNTs were damaged or had open ends (Figure 4f). However, the majority of the BNNTs remained intact and undamaged.

### 3.2. BNNTs Dispersion in Aqueous Media

**Figure 5:**
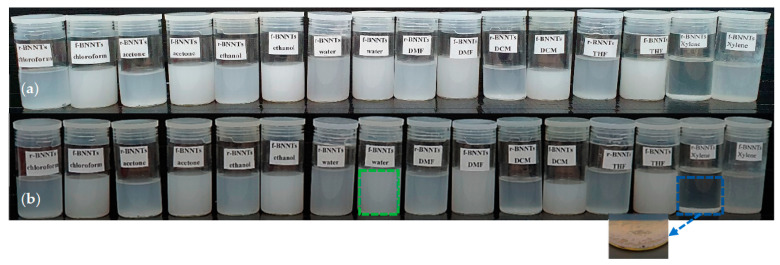
Dispersion of r-BNNTs and f-BNNTs in various solvents: (**a**) 12 h; (**b**) 48 h, inset: sedimentation in r-BNNTs after 48 h.

The dispersion of BNNTs in aqueous media was analysed by dispersing the r-BNNTs and f-BNNTs in eight types of solvents (deionised water, acetone, ethanol, chloroform, dichloromethane (DCM), dimethylformamide (DMF), tetrahydrofuran (THF) and xylene), which were chosen based on previous studies of the dispersion of BNNTs [55]. Figure 5 shows the dispersion of r-BNNTs and f-BNNTs in solvents. The dispersion was monitored for 12 h and 48 h. After 12 h, the r-BNNTs and f-BNNTs were cloudy in all of the solvents, while the r-BNNTs in xylene had undergone slight sedimentation, as shown in Figure 5a. After 48 h, the r-BNNTs were sedimented (inset Figure 5b) in most of the solvents, but were slightly cloudy in ethanol and water (Figure 5b). The f-BNNTs were cloudy and stable in all of the solvents except xylene after 48 h (Figure 5b). Thus, it was evident that the synthesised BNNTs, with further purification and functionalisation, were more stable in various solvents compared to r-BNNTs.

### 3.3. FTIR Spectrum

**Figure 6:**
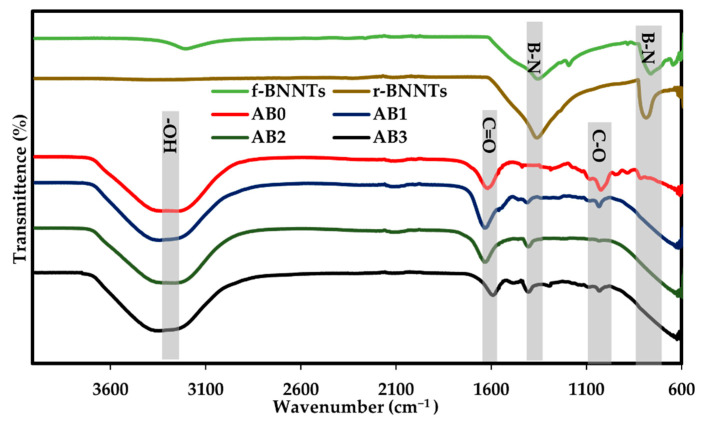
FTIR spectra of BNNTs and AB ink solutions.

The FTIR spectrum of the r-BNNTs and f-BNNTs are shown in Figure 6. The spectrum of the as-synthesised BNNTs showed two firm peaks at 1357 cm^−1^ and 798 cm^−1^, which correspond to primary and secondary absorption bands of h-BN. The intense peak at 1357 cm^−1^ was related to the transverse optical mode of h-BN along with longitudinal or tube axis vibrations. At the same time, a weak peak at 798 cm^−1^ resulted in out-of-plane vibrations. Furthermore, a solid, intense peak of f-BNNTs was observed at 3200 cm^−1^ that could be attributed to the stretching vibration of N-H reactions. The spectra of the H_2_O_2_ revealed a band at approximately 3200 cm^−1^ in f-BNNTs that may be attributed to -OH groups [50,56]. An additional peak in f-BNNTs can be observed due to the carbohydrate attachments (C-O) at 1200 cm^−1^. These features indicate that the -OH and C-O containing chains are bonded to the BNNTs [56]. Thus, it was indicated that after H_2_O_2_ treatment, the BNNTs had been effectively functionalised. To further confirm that the produced products were BNNTs, the FTIR spectrum was compared with BNNT spectra reported by Wang et al. [50], Wang et al. [44] and Budy et al. [51]. The produced BNNTs and f-BNNTs displayed bands (Figure 6) similar to those reported in the literature, confirming that the synthesised and functionalised products were BNNTs.

The AB0 ink displayed prominent peaks of O-H (3367 cm^−1^), C=O (1718 cm^−1^) and C-O (1029 cm^−1^) groups, respectively. The relatively small peaks at 1235 cm^−1^ (C-C-H and O-C-H), 1168 cm^−1^ (C-O-C and C-OH) and 1076 cm^−1^ (C-O and C-C) bands were attributed to the vibration of the pyranose rings. The spectrum of AB1, AB2 and AB3 revealed the major high wavenumber of in-plane B-N stretching at 1360 cm^−1^ to have no significant shift corresponding to BNNTs. Thus, the obtained BNNTs reinforced Alg ink revealed a homogeneous dispersion of BNNTs in a polymer matrix with well-defined BNNTs concentration. The peaks of AB hydrogels were further compared with Wang et al.’s work [50], reporting the spectrum of BNNTs-alginic acid solutions.

### 3.4. Printability of Alg-BNNTs Ink

**Figure 7:**
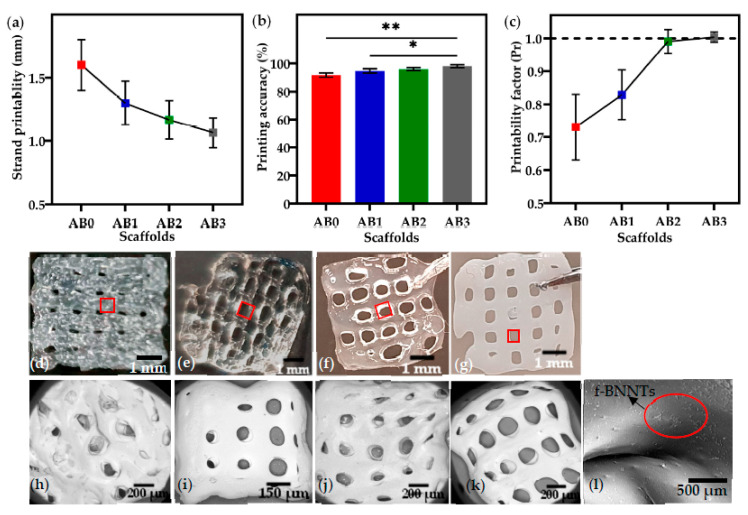
(**a**) Strand printability of ink solutions; (**b**) printing accuracy of ink solutions (** *p* < 0.01, * *p* < 0.05; *n* = 3); (**c**) printability factor (Pr) of ink solutions; images of scaffold (**d**) AB0; (**e**) AB1; (**f**) AB2; (**g**) AB3; SEM images of scaffold topography (**h**) AB0; (**i**) AB1; (**j**) AB2; (**k**) AB3; (**l**) AB3 (higher magnification with BNNTs dispersion in scaffold).

The ink was extruded into 0° and 90° strands with a single layer, and the strand printability for all ink under the printing conditions (Table 1) was studied. From Figure 7a–c, it can be observed that the presence of f-BNNTs improved printing outcomes. The strands of AB0 (1.6 ± 0.3 mm) and AB1 (1.3 ± 0.2 mm) were wider than the designed model, with a width of 1 mm, while AB2 (1.1 ± 0.06 mm) and AB3 (1 ± 0.02 mm) were observed to be near perfect strands printed with similar dimensions as the designed strands.

The results showed that increasing the concentration of f-BNNTs resulted in a more accurate printing (Figure 7b). The AB2 and AB3 printed scaffolds showed dimensions approximately equal to the designed model, with 98.5% and 99.0% accuracy, respectively. The printing accuracy of AB0 and AB1 was observed to be less than that of AB3. As with strand thickness and printing accuracy, the presence of BNNTs significantly improved printability (Figure 7c). AB0 showed poor printability (Pr ≈ 0.7). In comparison, the AB3 scaffolds had almost perfectly square pores with good interconnectivity, resulting in a printability factor near 1. The scaffold images are shown in Figure 7d–g with the pores highlighted in red square. The SEM images of pore structures of the scaffolds surface topography are shown in Figure 7h–k. It can be seen that the f-BNNTs (red circle) were uniformly dispersed in Alg, and the printed scaffolds without agglomeration were observed at higher magnification as shown in Figure 7l.

### 3.5. Contact Angle Measurement

**Figure 8:**
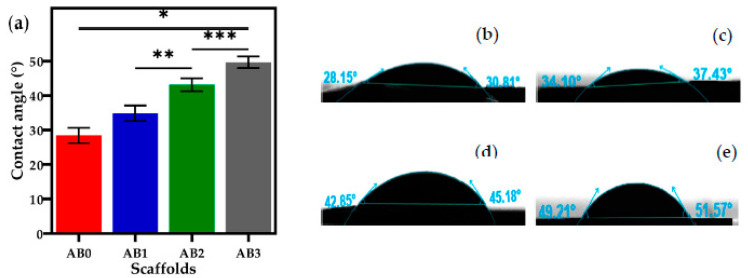
(**a**) Contact angle measurements of scaffolds (* *p* < 0.05, ** *p* < 0.01, *** *p* < 0.001; *n* = 3); sessile drop images (**b**) AB0; (**c**) AB1; (**d**) AB2; and (**e**) AB3.

The contact angle measurements of scaffolds are shown in Figure 8a and the sessile drop images of scaffolds are shown in Figure 8b. The contact angles of AB0 and AB1 were determined to be 28.45 ± 2.3° and 34.88 ± 2.0°. For higher BNNTs concentrations, such as AB2 and AB3, the angles increased significantly to 43.18 ± 2.0° and 49.69 ± 1.6°, respectively. The fact that the angles determined were less than 90° signifies that both surfaces showed good wetting behaviour. Furthermore, it was clear that an increase in f-BNNT concentration reduced the interfacial tension of the surface. Hence, the scaffolds were moderately hydrophilic and may have decreased the infusion of water molecules and degradation. Nevertheless, the f-BNNTs with -OH group allowed water to be absorbed upon exposure to the aqueous medium.

### 3.6. Mechanical Properties

**Figure 9:**
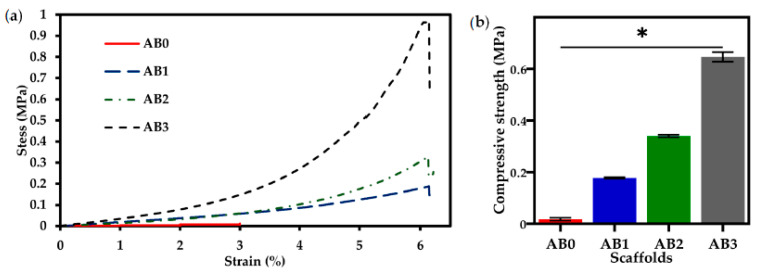
(**a**) Compressive stress vs. strain graph; and (**b**) compressive strength of the scaffolds (* p < 0.05; *n* = 3).

Figure 9a shows the compressive stress vs. strain curves for the AB scaffolds. Compressive strength was found to increase with increasing concentration of f-BNNTs. AB0 was compressed by up to 3% of strain, while AB1, AB2 and AB3 were compressed by 6%. With the increase of f-BNNTs loading, the stress rate applied on the scaffolds increased from 0.2 MPa to 0.9 MPa. The scaffold’s compressive strength is shown in Figure 9b. AB0 (0.01 ± 0.02 MPa) shows less compressive strength than AB3 (0.6 ± 0.2 MPa). Thus, it can be seen that with the addition of f-BNNTs, the hydrogen bonds formed between Alg chains reduced, and the interaction between matrix and filler was enhanced, leading to an increase in the compressive strength of the scaffolds.

### 3.7. In Vitro Cell Viability Tests

**Figure 10:**
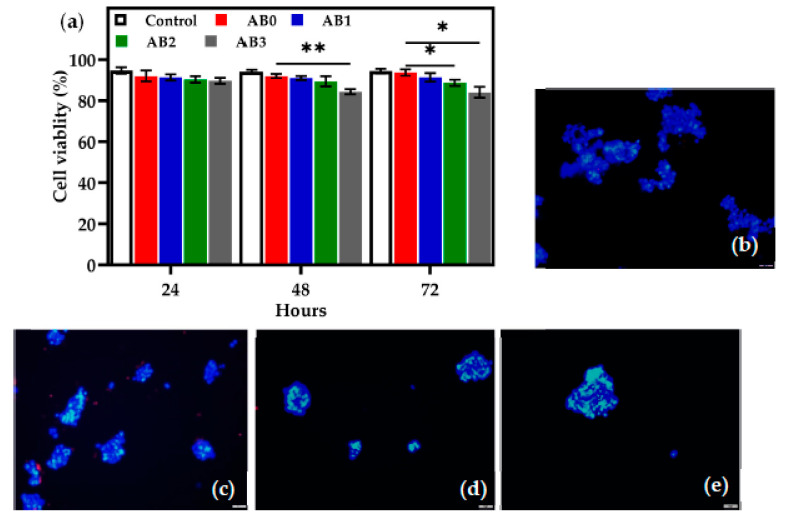
(**a**) Cell viability of control and AB scaffolds (* *p* < 0.05, ** *p* < 0.01; *n* = 3); fluorescent images of (**b**) AB0; (**c**) AB1; (**d**) AB2 and (**e**) AB3.

The viability of the HEK 293T in the AB0, AB1, AB2 and AB3 scaffolds was determined after 24, 48 and 72 h (Figure 10a). After 24 h, the cell viability rate for AB0, AB1, AB2 and AB3 was recorded to be 92 ± 0.5%, 91 ± 0.3%, 90 ± 0.3% and 89 ± 0.6%, respectively. After 48 h, AB0, AB1 and AB2 do not show any major variation, while the viability rate of AB3 decreased (84 ± 0.3%). After 72 h, the viability rate of AB0 (93 ± 0.6%) and AB1 (91 ± 0.3%) increased, but AB2 (88 ± 0.6%) and AB3 (84 ± 1%) showed a slightly reduced cell viability.

Overall, the cell viability rate of AB0 was the highest, followed by AB1, AB2 and AB3. There were significant differences in cell viability between AB1 and AB3 as presented in Figure 10a. The results indicate that the increase in the concentration of f-BNNTs influenced the viability of the HEK 293T cells. Additionally, viability was further investigated using the LIVE/DEAD^®^ viability kit. As shown in Figure 10b–e, introducing AB scaffolds to HEK 293T cells caused cell viability to decrease when the concentration of f-BNNTs increased in Alg after 24 h.

## 4. Discussion

In recent years, hydrogel-based inks have been utilised for extrusion-based bioprinting to produce complex tissue models and organ structures [45,46]. For example, studies have been carried out to reproduce the zonal structure of cartilage by enhancing the scaffold design [45,46]. Other studies have reported using hydrogel inks incorporated with cells to produce human-scale functional tissues according to patient-specific tissue geometry [45,46]. The current study aimed to develop a hydrogel-based ink with enhanced physicochemical properties with Alg as the main component and f-BNNTs as the reinforcement. First, the r-BNNTs were synthesised with a diameter ranging from 10 to 300 nm, composed of both bamboo-shaped and quasi-cylindrical morphologies (Figure 5a–c). According to Lee et al. [57], the functionalisation of the produced BNNTs is essential for application in areas of biomedicine. Additionally, the r-BNNTs were hydrophobic and formed inhomogeneous aggregates in aqueous media [57]. Thus, the r-BNNTs were further purified and functionalised to obtain good dispersion of BNNTs in the aqueous solutions. It was observed that the f-BNNTs were stable in aqueous media after 48 h without evident precipitation, compared to the r-BNNTs (Figure 5).

Second, a f-BNNT reinforced Alg hydrogel-based ink solution was successfully produced to print porous scaffolds using extrusion bioprinting. For 3D bioprinting, an ideal bioink must possess good printability and mechanical properties to produce a tissue’s internal structure and external shape. These properties contribute to supplements and metabolic activity of the cells during and after tissue development. In Table 2, the strand thicknesses, printing accuracy and printability factor of the highest concentration of f-BNNTs indicated dimensions almost identical to those of the designed scaffold. AB3 showed ideal printability properties with an increase in compressive strength.

The printed scaffolds were produced under continuous ink extrusion with well-interconnected pores [58]. The interconnected pore structures in the AB scaffolds showed a significant difference compared to the scaffolds without BNNTs. Printing of AB ink with higher BNNT concentration resulted in thinner strands and square pores (Table 2), likely because of molecular interactions between Alg and BNNTs. Zhang et al. [52] demonstrated that the printability of Alg-incorporated graphene oxide resulting in improved printability and scaffold fidelity of graphene reinforced Alg. Furthermore, it should be noted that the strands’ fidelity was enhanced due to the Ca^2+^ being crosslinked immediately after printing. Serafin et al. [59] investigated hydrogel ink that was formulated using alginate, gelatin and carbon nanofibers. The hydrogels with increased concentrations of carbon nanofibers showed improved mechanical and printability properties. Habib et al. [60] developed alginate with carboxymethyl cellulose hydrogel for extrusion printing. The outcomes displayed good shape fidelity with the addition of carboxymethyl cellulose. Similarly, in this study, the addition of BNNTs improved the strand thickness, printing accuracy and pore connectivity of scaffolds.

It was highly preferred that the scaffolds seeded on cells should not be soluble in water since they are required to offer mechanical assistance to the cells until tissue regeneration is complete. Hydrophobicity of scaffolds contributes to better adhesion of cells and proteins on their surfaces. Therefore, the hydrophobicity of the scaffold is a vital parameter for cell adherence. The AB0 scaffolds have less water resistance due to the hydrophilic nature of Alg (Table 2). AB1, AB2 and AB3 showed an increase in water resistance (Table 2). It can be noted that the scaffolds that had high contact angle measurement were more hydrophobic and could be used for cell adhesion and proliferation studies. According to Holler et al. [61], the contact angle of pure Alg was less (15°) compared to Alg crosslinked with CaCl_2_ (30°). Thus, it was evident that the crosslinked Alg aided in increasing the water-resistant contact angle of AB0 (24.87 ± 5°). Likewise, Lavric et al. [62] reported on the contact angle of nanocomposite film with combination Alg and cellulose nanocrystals. The results showed that the highest contact angles were observed in high concentrations of cellulose nanocrystals. The present study found similar results, with the incorporation of BNNTs in Alg resulting in an increase in the water resistance, which is vital for cell adherence and protein absorption during biomedical applications.

In this study, the thermal properties of Alg and Alg incorporated with BNNTs scaffolds using TGA (see the Supplementary Materials) were compared. The results showed that degradation and carbonisation processes occurred at marginally elevated temperatures in AB with varied concentrations of BNNTs compared to the AB0 scaffold. This indicated that BNNTs enhanced the thermal stability of Alg and retarded the pyrolysis of the scaffolds [63,64]. It might be connected with the fact of BNNTs limiting the change of Alg polymeric chains, impeding the thermal decomposition process and improving the energy required for thermal decomposition [63,64]. Lastly, it was evident that the thermal properties of Alg were affected by intercalation with BNNTs and subsequent crosslinking with CaCl_2_.

Mechanical properties are essential characteristics for biomedical applications, especially when the biomaterials are utilised in tissue engineering applications. Adding BNNTs to Alg substantially increased its mechanical strength. The results showed that adding f-BNNTs to Alg led to an increase in the compressive strength (0.01 to 0.6 MPa) of the scaffolds (Table 2). This could be attributed to the interactions of functional groups on the edges of the BNNTs and the hydroxyl groups on the Alg backbone that support the load transfer from matrix to filler, increasing the compressive strength and taking up more compressive stress before complete failure. According to Choe et al. [28], bioinks formulated with graphene oxide reinforced Alg demonstrate higher mechanical strength than graphene oxide-free inks due to strong hydrogen bonding between polymer chains and graphene oxide. Furthermore, it is known that many human tissues, such as tendons and blood vessels, tend to bear strong mechanical force under physiological conditions [29]. Therefore, tissues regenerated using biomaterials have substantial mechanical properties that natural polymers cannot achieve. Typically, the strength of human tissues ranges from 0.01 MPa to 150 MPa [65,66]. Thus, the current study confirms that the mechanical properties of the produced AB scaffolds are (Table 2) suitable for printing tissue structures with good pore interconnectivity.

Another important factor for ideal ink to be used in bioprinting is the biocompatibility of the materials. In this study, HEK 293T cell interaction with AB scaffolds was compared with scaffolds without BNNTs. After 24 h, cell viability was approximately 90% for all scaffolds, with a higher rate in control and AB0. However, after 48 h, the cells treated with AB1, AB2 and AB3 showed decreased cell viability compared to the control and AB0. The decrease in viability rate could be a result of the consequences of cell interaction with BNNTs, causing low toxicity. As time passed, a significant difference was observed between AB0 and AB3, showing a lower viability level for the latter. Furthermore, the ReadyProbes staining disclosed that both AB0 and AB with varied concentrations of BNNTs had an adequate level of biocompatibility (Figure 10b–e). The staining displayed that the number of viable cell nuclei was similar across all scaffolds. According to Chen et al. [67], BNNT concentration of 100 mg/mL cultured for four days showed that the BNNTs were non-toxic to HEK 293T cells. In contradiction, Horvath et al. [68] found that BNNTs (2–20 µg/mL) were toxic to HEK 293T cells. The controversy surrounding BNNTs’ toxicity arises due to various studies employing nanotubes of different lengths [69,70]. Horvath et al. [68] used nanotubes longer than 10 µm, while Chen et al. [67] used nanotubes less than 6 µm. Thus, it is evident that the length of nanotubes plays a vital role in toxicity analysis. Overall, in the present study, the nanotube length was hundreds of micrometres, and the dosage was higher than in the literature studied; the results showed that BNNTs at higher concentrations displayed minimal toxicity to HEK 293T cells.

In this investigation, the inks of BNNTs reinforced Alg were successfully prepared and assessed. Ideal bioprinting ink needs to develop scaffolds under constant extrusion and maintain shape fidelity while demanding high cell viability to regenerate the tissue. The results found that AB3 exhibited good printability, water resistance and higher thermal stability and compressive strength than AB0. However, AB3 decreased the viability rate of HEK 293T compared to AB0.

Further work needs to be undertaken to establish whether Alg that incorporates BNNTs is a potential bioink for tissue engineering applications. The present investigation is limited to printability and mechanical properties of BNNT reinforced Alg ink. Considerably more work will need to be carried out to determine the optimum ink solution. Assessing ink viscosity through rheological experiments and measuring the shear stress of ink through extrusion using different nozzles will bring about a better understanding of printability. Additionally, the interaction between scaffolds and cell culture media components needs to be evaluated to determine degradation and swelling properties. Finally, it was evident that a higher concentration of BNNTs improved printability and mechanical properties, but it was also noted that a high dosage of BNNT scaffolds produced little toxicity to HEK 293T cells. Moreover, contradictory reports in the literature cause uncertainties about the biocompatibility of BNNTs. Thus, the biocompatibility of BNNTs needs further evaluation using other cell models to obtain a clear understanding of toxicity. Furthermore, incorporating cells into BNNTs reinforced Alg ink during printing, and further assessment of AB ink-composed cell extrusion could be helpful in future research. Future work will be directed at improving smoothness of BNNTs and investigating the longer-term effects of BNNTs on cell and tissue viability.

The 3D bioprinted BNNTs reinforced Alg scaffolds open a pathway for potential future research. The incorporation of BNNTs into Alg enhances the physiochemical properties of the scaffolds, with further evaluation of cellular activities to ensure the assembly of cell incorporated 3D structure printing. The approach can be extended to bioprinting of biomimetic structures which leads to the development of biological tissues such as skin, bone or cartilage structures [71].

## 5. Conclusions

In this paper, f-BNNTs reinforced Alg scaffolds were developed by bioprinting techniques; the effects of printability and the physiochemical and biocompatibility properties of each scaffold were studied. AB3 scaffolds presented good printability and physicochemical properties for tissue regeneration post-printing. However, AB3 displayed minimal toxicity compared to the pure Alg scaffold. Thus, further study is required to investigate the effects of low concentrations of BNNTs on cell proliferation and differentiation. In addition, cell-laden tissue structures with HEK 293T cells and different functional cells should be studied to determine the physiological properties of cells after extrusion.

To sum up, the stated outcomes can be considered to advance the fields of tissue engineering applications of BNNTs and hydrogel-based ink solution for 3D bioprinting since they expand the biofabrication window by application of BNNTs reinforced Alg hydrogel ink for developing biomimetic ink solution and tissue structures.

## Figures and Tables

**Table 1:** The ink composition of nanocomposites hydrogel-based ink solutions and the parameters used for 3D bioprinting parameters.

Hydrogel-Based Ink	Alg (A) (*w*/*v*%)	f-BNNTs (B) (*w*/*v*%)	Nozzle Gauge (G)	Crosslinking Time (min)	Pressure (kPa)
AB0	5	0	27	15	80 ± 1
AB1	5	0.05	27	15	85 ± 2
AB2	5	0.075	27	15	93 ± 2
AB3	5	0.1	27	15	109 ± 4

**Table 2:** Mean ± SD data for AB scaffolds.

Sample	Pressure (kPa)	Strand Thickness (mm)	Printing Accuracy (%)	Printability (Pr)	Contact Angle (°)	Compressive Strength (MPa)
AB0	80 ± 1	1.6 ± 0.2	91 ± 3	0.73 ± 0.12	28.45 ± 2.3	0.01 ± 0.2
AB1	85 ± 2	1.3 ± 0.3	94.5 ± 2	0.79 ± 0.05	34.88 ± 2.0	0.17 ± 0.1
AB2	93 ± 2	1.1 ± 0.2	96.2 ± 2	0.99 ± 0.01	43.19 ± 2.0	0.34 ± 0.1
AB3	109 ± 4	1.0± 0.3	98.3 ± 2	1 ± 0.01	49.69 ± 1.6	0.64 ± 0.1

## Supplementary Materials

The following are available online at https://www.mdpi.com/article/10.3390/polym14030486/s1, Figure S1: (**a**) TGA curves and (**b**) DTG of AB scaffolds.
