# Supplementary material for: Extrusion-Based Bioprinted Boron Nitride Nanotubes Reinforced Alginate Scaffolds: Mechanical, Printability and Cell Viability Evaluation
Source: Polymers (Basel). 2022 Jan 26;14(3):486. doi: 10.3390/polym14030486 (PMC8839966; doi:10.3390/polym14030486)
Supplement: Supplementary file 1 [file polymers-14-00486-s001.zip › Alg-BNNTs_ Manuscript_ Revised_Supplementary Material.pdf]

polymers MDPI

Supplementary Material

Extrusion Based Bioprinted Boron Nitride Nanotubes Reinforced Alginate Scaffolds: Mechanical, Printability and Cell Viability Evaluation

Akesh Babu Kakarla [1], Ing Kong [1,*], Cin Kong [2] and Helen Irving [3]

[1] School of Computing, Engineering and Mathematical Sciences, La Trobe University, Bendigo, VIC 3552, Australia; A.Kakarla@latrobe.edu.au
[2] Department of Biomedical Sciences, Faculty of Science and Engineering, University of Nottingham Malaysia Campus, Semenyih 43500, Selangor, Malaysia; kong_cin@hotmail.com
[3] Department of Rural Clinical Sciences, La Trobe Institute for Molecular Sciences (LIMS), Bendigo, VIC 3552, Australia; H.Irving@latrobe.edu.au
* Correspondence: I.Kong@latrobe.edu.au

Citation: Kakarla, A.B.; Kong, I.; Kong, C.; Irving, H. Extrusion-Based Bioprinted Boron Nitride Nanotubes Reinforced Alginate Scaffolds: Mechanical, Printability and Cell Viability Evaluation. *Polymers* **2022**, *14*, 486. https://doi.org/10.3390/polym14030486

Academic Editor: Young-Sam Cho

Received: 6 January 2022
Accepted: 23 January 2022
Published: 26 January 2022

Publisher's Note: MDPI stays neutral with regard to jurisdictional claims in published maps and institutional affiliations.

Copyright: © 2022 by the authors. Licensee MDPI, Basel, Switzerland. This article is an open access article distributed under the terms and conditions of the Creative Commons Attribution (CC BY) license (https://creativecommons.org/licenses/by/4.0/).

1. Thermal properties

TGA and DTG curves of AB0, AB1, AB2 and AB3 crosslinked with $CaCl_2$ are presented in Figure S1 (a) and (b). It can be seen from Figure S1 (a), each TGA curve displayed four degradation stages. The first stage, up to 100 °C, was due to the loss of water molecules. The second step, which occurred from approximately 110 °C to 230 °C, was due to the degradation of oxygenated functional groups of Alg. The third step, ranging from around 230 °C to 350 °C, represents the removal of side chains of hydrogel molecules. The last step began at 350 °C, which resulted in the degradation of polymer backbones into ashes. It was also noted that the thermal stability of Alg increased with an increase of f-BNNT loading.

Further, DTG curves for all the scaffolds crosslinked with $CaCl_2$ were shown in Figure S1 (b). At about 80 to 230 °C, all the scaffolds exhibited the first weight loss of approximately 3%. Between 230 °C and 350 °C, the major weight loss peak of the scaffolds was observed. The sharper peak for the AB0 suggests that the moisture content was higher than AB1, AB2 and AB3. Therefore, it can be inferred that an increase in the concentration of f-BNNTs reduced the absorption of water, displaying the interference of f-BNNTs decreases the polarity and hygroscopicity of the Alg. At 240 °C, scaffolds continued to be decomposed with peak weight loss of approximately 30%. AB0 (32%) displayed highest decomposition, followed by AB1 (30%), AB2 (29%) and AB3 (28%) respectively. As the heat transfer rate increased, the decomposition rate of AB1, AB2 and AB3 were lower compared to AB0 as a result of increasing in f-BNNTs. From 250 °C to 630 °C, the weight of scaffolds was reduced to about 60%. Between 630 to 730 °C, a noticeable weight loss peak denotes the left-over deposits into char. After that, the degradation rate remained constant.

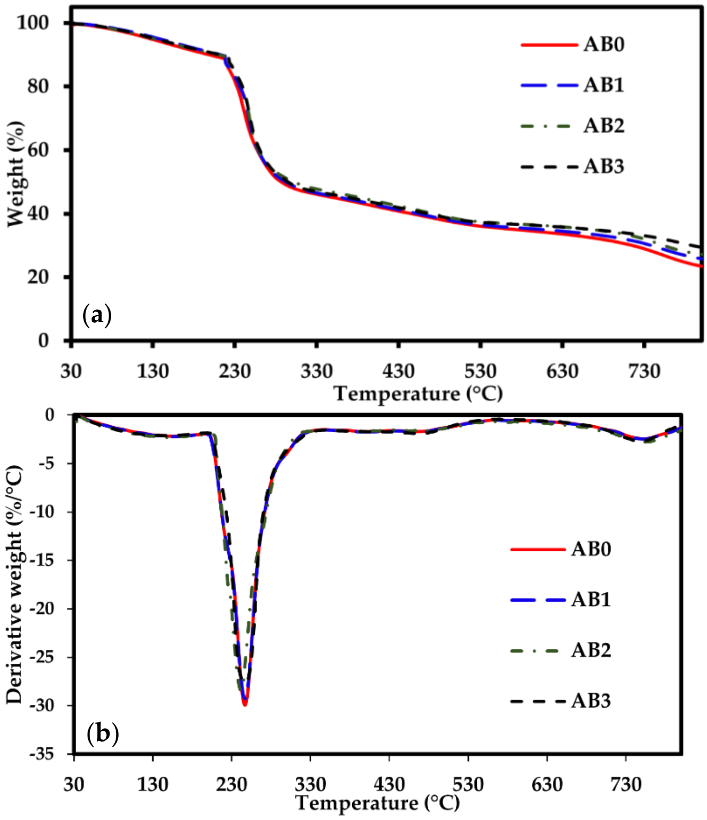

Figure S1. (**a**) TGA curves; and (**b**) DTG of AB scaffolds.
